# GOLPH3 overexpression correlates with poor response to neoadjuvant therapy and prognosis in locally advanced rectal cancer

**DOI:** 10.18632/oncotarget.12008

**Published:** 2016-09-13

**Authors:** Kunli Zhu, Qianqian Zhao, Jinbo Yue, Pengyue Shi, Hongjiang Yan, Xiaoqing Xu, Renben Wang

**Affiliations:** ^1^ Department of Radiation Oncology, Shandong Cancer Hospital, Affiliated to Shandong University, Jinan, China; ^2^ School of Medicine and Sciences, University of Jinan-Shandong Academy of Medical Sciences, Jinan, China

**Keywords:** GOLPH3, neoadjuvant chemoradiotherapy, rectal cancer, tumor response, survival

## Abstract

Neoadjuvant chemoradiotherapy (nCRT) combined with surgery is a standard therapy for locally advanced rectal cancer (LARC). The aim of this study was to assess the expression of GOLPH3 (Golgi phosphoprotein 3), a newly found oncogene, in LARC as well as its relationship with nCRT sensitivity and prognosis. We retrospectively analyzed 148 LARC cases receiving nCRT and total mesorectal excision (TME). Immunohistochemistry was used to assess GOLPH3 and mTOR (mammalian target of rapamycin) in tumor tissues. Then, the associations of GOLPH3 with pathological characteristics and prognosis of rectal cancer were assessed. The 148 cases included 77 with high GOLPH3 expression (52.03%), which was associated with tumor invasive depth and lymphatic metastasis. Cases with high GOLPH3 expression had 2.58 and 2.71 fold higher local relapse and distant metastasis rates compared with the low expression group. Correlation analyses showed that GOLPH3 was an independent indicator for judging tumor down-staging and postoperative TRG (tumor regression grade), indicating it could predict nCRT sensitivity. In addition, GOLPH3 expression was associated with mTOR levels. Multiple-factor analysis indicated that GOLPH3 was an independent prognosis indicator for 5 year-DFS (disease free survival) and OS (overall survival) in LARC. These results reveal that GOLPH3 is an independent predictive factor for nCRT sensitivity and prognosis in LARC, with a mechanism related to mTOR.

## INTRODUCTION

Morbidity and mortality of colorectal cancer is steadily increasing, which severely threatens human health. This tumor is the third cause of death from all malignancies [1]. In the past 30 years, therapies for rectal cancer have gained great improvements. Indeed, total mesorectal excision (TME) and neoadjuvant chemoradiotherapy (nCRT) greatly decrease local relapse rate and increase patient survival, and are considered standard therapies for locally advanced rectal cancer (LARC) [2–4]. However, markedly different therapeutic effects are obtained for various nCRT modalities; some cases can achieve pathological complete response (pCR), while others are not sensitive or even resistant. Therefore, sensitivity genes should be urgently identified to predict the effect of nCRT on rectal cancer. This would allow accurate individualized therapy.

GOLPH3 (Golgi phosphoprotein 3) belongs to the first class of Golgi proteins [5]. It is a highly preserved protein and an effector for phosphatidylinositol-4-monophosphate (PI4P) in the Golgi apparatus [6]. It is demonstrated that GOLPH3 plays a very important role in regulating cell division, mitochondrial mass, and DNA damage [7–10]. Furthermore, GOLPH3 activates the mTOR (mammalian target of rapamycin) pathway, Scott et al. reported [11], which is critical in the regulation of cell proliferation, growth and activity [12]. Recent studies indicated that GOLPH3 was highly expressed in malignant tumors, including colorectal, breast, renal, pancreatic, and non-small cell lung cancers, and closely associated with clinical staging and poor prognosis of the tumor [13–17].

Two reports assessing GOLPH3 in rectal cancer have been published. Guo et al. showed that GOLPH3 was highly expressed in rectal cancer (53.2%), significantly higher than the value obtained for the normal tissue (24.2%). In addition, patients with high GOLPH3 expression had poorer prognosis [13]. Wang et al. demonstrated that GOLPH3 was highly expressed in colorectal cancer (43.1%), higher than in the surrounding normal tissue (13.3%) [18]. Furthermore, GOLPH3 overexpression was shown to promote the apoptotic effect of 5-Fu. Taken together, findings suggested that GOLPH3 could be used as an important indicator for predicting sensitivity to 5-Fu [18]. However, there is no report available to assess the relationship between GOLPH3 and efficacy of nCRT for rectal cancer.

The aim of this study was to assess GOLPH3 expression in rectal cancer, and determine its associations with clinical pathological characteristics and prognosis, evaluating its value as a potential independent predictive indicator for nCRT sensitivity and prognosis. Meanwhile, mTOR expression was quantitated to explore the underlying mechanism.

## RESULTS

### Patient characteristics

The clinical pathological characteristics of the 148 LARC patients are listed in Table 1. There were 89 (60.14%) males and 59 (39.86%) females, with 81 (54.73%) patients above 65 year-old and 67 (45.27%) below 65. Average patient age was 63.2 years. The lump was located at 6 cm within the anal verge in 57 (38.51%) cases, and beyond 6 cm in the remaining 91 (61.49%) patients. Postoperative pathological results indicated 82 (55.41%) cases with high tumor tissue differentiation, and 66 (44.59%) with low differentiation. Among the 148 cases, 80 (54.05%) and 68 (45.95%) had cT3 and cT4 disease stages, respectively; 54 (36.49%) cases were in cN0 stage, and 94 (63.51%) in cN+ stage.

**Table 1:** Correlations between GOLPH3 expression and clinicopathological parameters

Clinicopathological parameters	Cases (*n* = 148)	GOLPH3 expression		*P* value
		Low (71)	High (77)	
Gender				
Male	89	41	48	0.569
Female	59	30	29	
Age (years)				
< 65	67	35	32	0.345
≥ 65	81	36	45	
Histology				
Differentiated	82	47	35	0.011
Undifferentiated	66	24	42	
Distance from anal verge(cm)				
< 6	57	29	28	0.576
≥ 6	91	42	49	
CEA(ng/ml)				
< 3.4	70	41	29	0.014
≥ 3.4	78	30	48	
Clinical Tumor status				
cT3	80	45	35	0.029
cT4	68	26	42	
Clinical Node status				
cN0	54	32	22	0.037
cN+	94	39	55	
Recurrence				
Negative	105	59	46	0.002
Positive	43	12	31	
Distant metastasis				
Negative	122	64	58	0.018
Positive	26	7	19	

Abbreviations: GOLPH3, Golgi phosphoprotein 3; CEA, carcinoembryonic antigen.

### Expression of GOLPH3 in tumor and normal tissues and its associations with clinicopathological parameters of LARC

**Figure 1:**
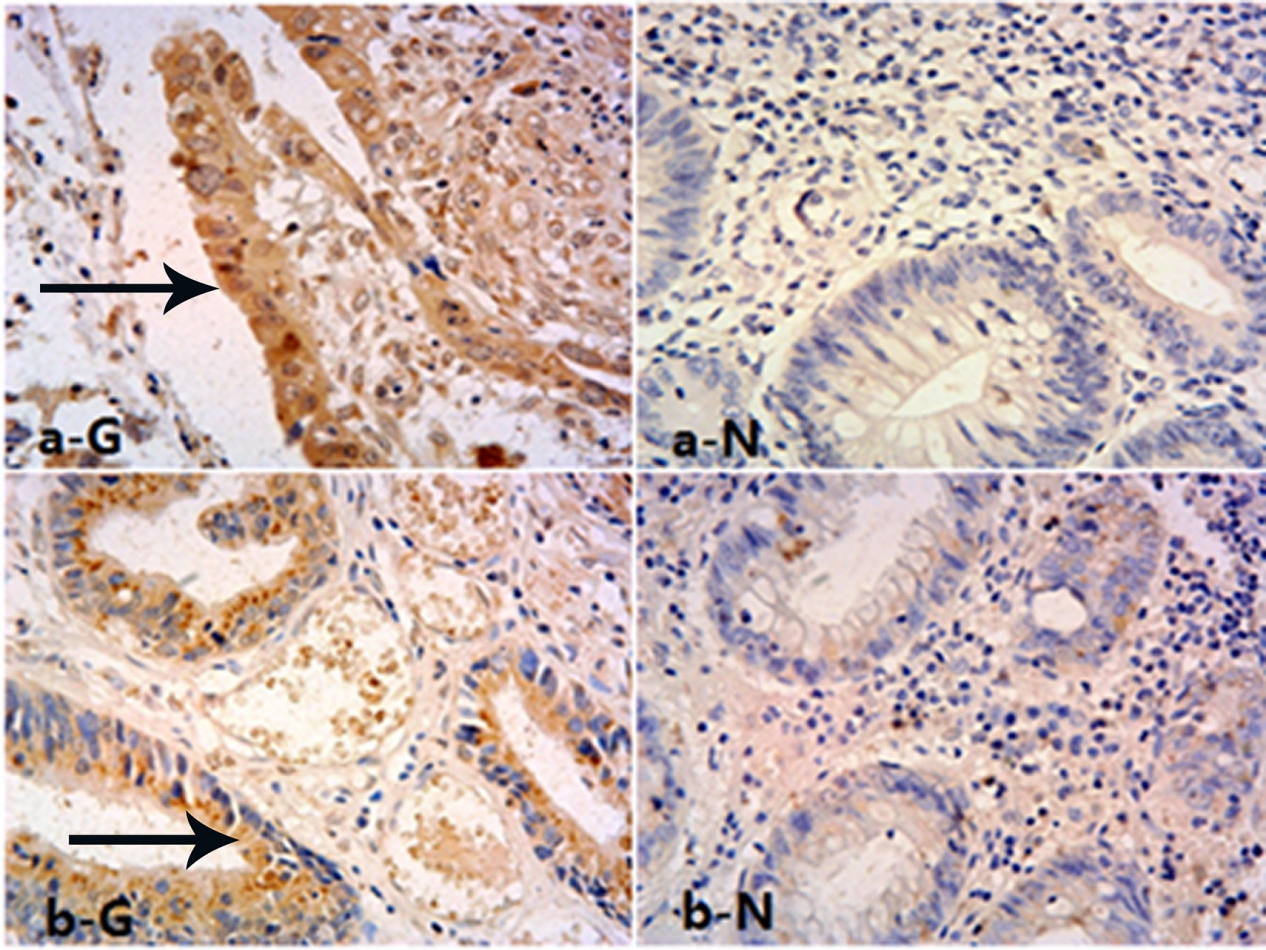
Expression of GOLPH3 in rectal tissues Immunohistochemical staining for GOLPH3 in rectal cancer cells cytoplasm (T: Tumor, High) and matched normal tissues (N: Normal, Low), magnification is 400×.

The associations of GOLPH3 expression and LARC clinical pathological characteristics are shown in Table 1. GOLPH3 was highly expressed in rectal cancer cell cytoplasm, with 77 (52.03%) cases showing high expression. However, low expression was found in normal surrounding rectum tissues, with only 11 of 87 (12.64%) cases showing high expression. The difference was statistically significant (*P* < 0.001) (Figure 1). In addition, high GOLPH3 expression was associated with tumor differentiation (*P* = 0.011), increased serum CEA (*P* = 0.014), tumor invasion depth (T staging) (*P* = 0.029) and lymphatic metastasis (*P* = 0.037), but not with gender, age and tumor location. Among the 77 patients with high GOLPH3 expression, 31 cases had local relapse, while 19 patients showed distant metastasis. Of the 71 patients with low expression, 12 and 7 cases had local relapse and distant metastasis, respectively. These findings indicated significant differences in local relapse (*P* = 0.002) and distant metastasis (*P* = 0.018) rates between the two groups.

### Correlation between GOLPH3 expression and tumor response

According to the AJCC (Edition 7) standard, preoperative and postoperative pathological staging was performed to analyze the down-staging effect of nCRT. A total of 77 cases showed tumor down-staging among the 148 cases (52.03%). Meanwhile, TRG was used to assess the therapeutic effect of nCRT. There were 69 cases (46.62%) with TRG 3–4 (sensitivity to nCRT) and 79 (53.38%) with TRG 0–2 (non-sensitivity). Interestingly, low GOLPH3 expression tended to yield high sensitivity to nCRT: among the 71 patients with low expression, 44 cases showed tumor down-staging; meanwhile, 33 of the 77 cases with high expression showed down-staging (*P* = 0.020). And of the 71 low expression cases 41 showed TRG 3–4, with 28 such patients found among the 77 cases with high expression (*P* = 0.009). Besides, tumor down-staging and TRG 3–4 in patients with low CEA level and no lymph node metastasis were higher compared with values found in those with high CEA level and lymph node metastasis (*P* = 0.001 and 0.001; *P* = 0.001 and *P* = 0.001, respectively). As for tumor invasion depth, sensitivity to radiotherapy in cT3 stage patients was higher than in cT4 cases. *P* values for tumor down-staging and TRG were 0.035 and *P* = 0.027, respectively (Table 2).

**Table 2:** Correlations between clinicopathological parameters and tumor response in LARC

Parameters	Cases *n* = 148	TRG		*P* value	Tumor staging		*P* value
		Good response	Poor response		Down	Non-down	
Age(years)							
< 65	67	33	34	0.559	35	32	0.963
≥ 65	81	36	45		42	39	
Sex							
Male	89	43	46	0.612	50	39	0.214
Female	59	26	33		27	32	
Distance from anal verge							
< 6 cm	57	27	30	0.885	32	25	0.428
≥ 6 cm	91	42	49		45	46	
Histology							
Differentiated	82	37	45	0.684	40	42	0.378
Undifferentiated	66	32	34		37	29	
CEA(ng/ml)							
< 3.4	70	45	25	0.001	49	21	0.001
≥ 3.4	78	24	54		28	50	
Clinical Tumor status							
cT3	80	44	36	0.027	48	32	0.035
cT4	68	25	43		29	39	
Clinical Node status							
cN0	54	39	15	0.001	40	14	0.001
cN+	94	30	64		37	57	
GOLPH3							
Low	71	41	30	0.009	44	27	0.020
High	77	28	49		33	44	

Abbreviations: LARC, locally advanced rectal cancer; TRG, tumor regression grade ; CEA, carcinoembryonic antigen ; GOLPH3, Golgi phosphoprotein 3.

By multi-factor correlation regression analysis, it was found that low GOLPH3 expression was significantly associated with TRG (OR = 3.952; CI 1.655–10.327, *P* = 0.026) and tumor down-staging (OR = 2.951; CI 1.523–11.324, *P* = 0.021), suggesting GOLPH3 could independently predict sensitivity to nCRT in rectal cancer, with high sensitivity in the low expression group. Besides, tumor invasion depth and lymph node metastasis status also showed significant associations with TRG and tumor down-staging (0.039 and 0.034; 0.029 and 0.037, respectively) (Table 3).

**Table 3:** Multivariate analysis for tumor response of nCRT in LARC

Parameters	Odds ratio	95% Confidence interval	*P* value
Good response			
CEA	1.871	0.834–3.981	0.182
cT	2.762	1.112–6.356	0.039
cN	2.654	1.136–6.524	0.034
GOLPH3	3.952	1.655–10.327	0.026
Down staging			
CEA	1.961	0.864–4.125	0.352
cT	2.638	1.109–6.356	0.029
cN	2.456	1.694–5.324	0.037
GOLPH3	2.951	1.523–11.324	0.021

Abbreviations: nCRT, neoadjuvant chemoradiotherapy; LARC, locally advanced rectal cancer ; cT, clinical Tumor status; cN, clinical Node status; CEA, carcinoembryonic antigen; GOLPH3, Golgi phosphoprotein 3.

### The relationship between GOLPH3 and mTOR expression

**Figure 2:**
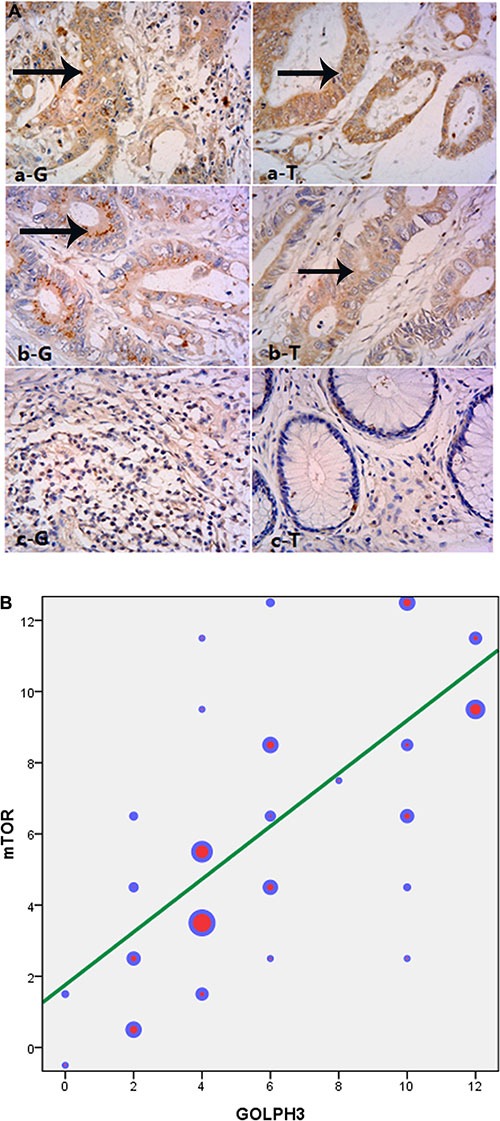
The expression of GOLPH3(G) and mTOR (T) were highly consistent in LARC cases (**A**) Immunohistochemical staining for GOLPH3 and mTOR in rectal cancer cells cytoplasm, magnification is 400×, a, b, c were three typical cases of high, medium and low expression. (**B**) mTOR was also highly expressed in rectal cancer tissues, and 81 cases with high expression (81/148, 54.73%). There were 53 cases with high mTOR expression among the 77 patients showing high GOLPH3 expression, and correlation analysis was done(*r* = 0.745, *P* < 0.001). The color represented the number of each gene expressed respectively.

In this study, mTOR was also highly expressed in rectal cancer tissues, and 81 cases with high expression (81/148, 54.73%) were obtained, which was consistent with high GOLPH3 expression. There were 53 cases with high mTOR expression among the 77 patients showing high GOLPH3 expression; meanwhile, 43 cases with low mTOR expression were found among the 71 cases with low GOLPH3 expression. Then, the correlation between the two genes was tested by correlation analysis (*r* = 0.745, *P* < 0.001) (Figure 2).

### Association between GOLPH3 and 5 year-DFS and OS

**Figure 3:**
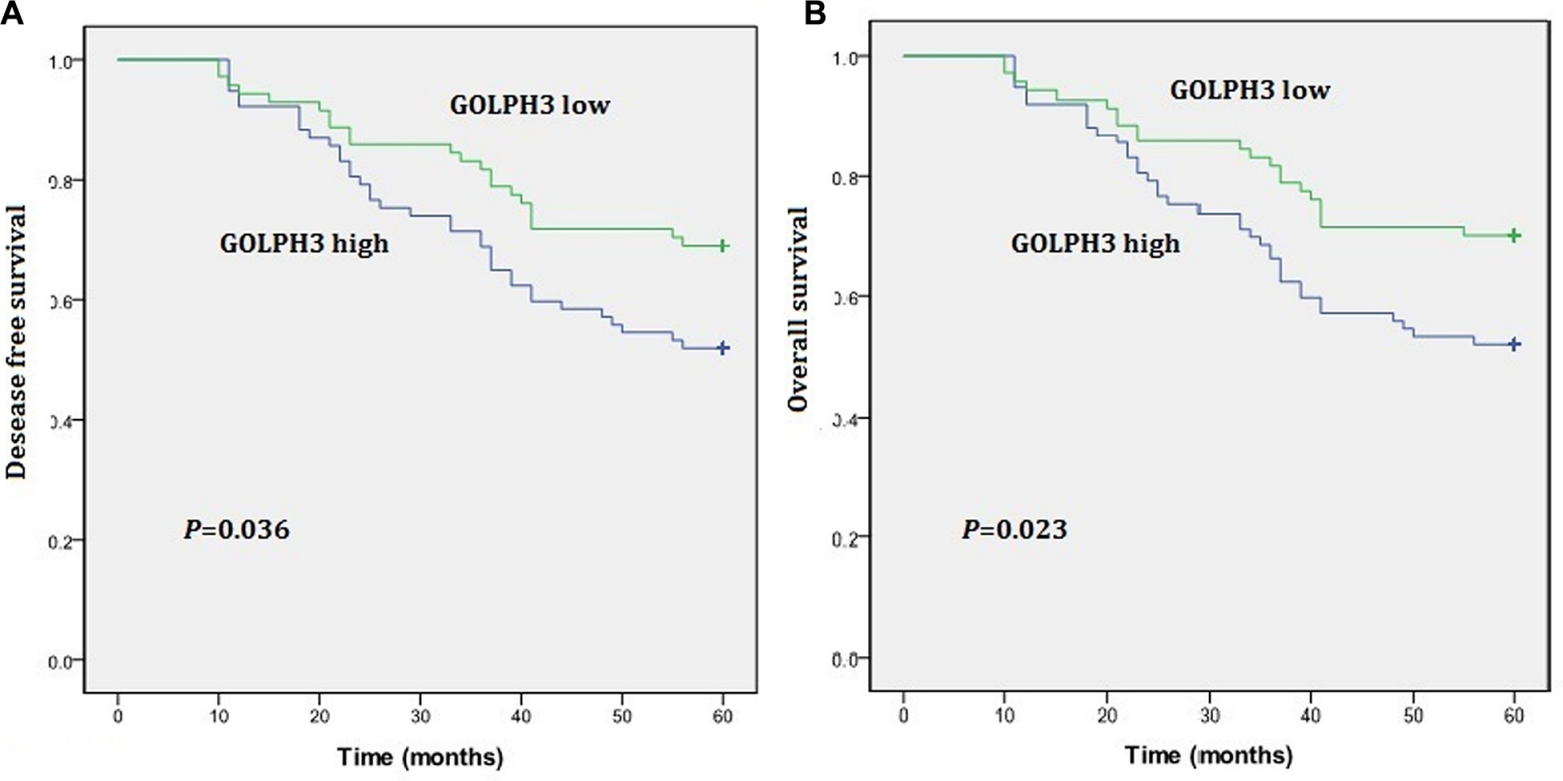
Kaplan-Meier estimates of disease-free survival (DFS) and overall survival (OS) rates in relation to GOLPH3 status (**A**) GOLPH3 high expression in rectal cancers correlate with a shorter DFS curve (*P* = 0.036). (**B**) GOLPH3 high expression in rectal cancers correlate with a shorter OS curve (*P* = 0.023).

Single factor survival analysis indicated that lymphatic metastasis status, tumor invasion depth, and GOLPH3 expression were associated with 5 year-DFS and OS (Table 4). Among the 148 LARC patients receiving nCRT, 5 year-DFS and OS in patients with high GOLPH3 expression were reduced compared with values obtained in the low expression group (*P* = 0.036 and 0.023, respectively) (Figure 3A and 3B). Multiple-factor analysis indicated that pN and pT were important prognosis factors for rectal cancer, as well as GOLPH3 expression. The prognosis of patients with high GOLPH3 expression was poorer compared with the low expression group. Indeed, GOLPH3 was shown to be an independent prognosis indicator for 5 year-DFS (HR = 2.624; 95% CI 1.235–6.541, *P* = 0.009) and OS (HR = 2.354; 95% CI 1.237–6.152, *P* = 0.039) in rectal cancer (Table 5).

**Table 4:** Univariate analysis between clinicopathological parameters and survival in patients with LARC

Parameters	Cases	5-year DFS, %	*P* value	5-year OS, %	*P* value
Age(years)					
< 65	67	65.3	0.611	73.2	0.536
≥ 65	81	61.2		70.9	
Sex					
Male	89	66.2	0.632	78.7	0.412
Female	59	61.8		66.8	
Distance from anal verge					
< 6 cm	57	61.1	0.395	71.5	0.712
≥ 6 cm	91	64.3		72.3	
Histology					
Differentiated	82	65.8	0.503	77.8	0.438
Undifferentiated	66	61.4		72.1	
CEA(ng/ml)					
< 3.4	70	62.3	0.342	63.5	0.312
≥ 3.4	78	58.1		57.6	
Pathological tumor stage					
pT0-2	62	69.2	0.039	81.8	0.041
pT3-4	86	57.1		60.2	
Pathological node stage					
pN0	88	75.6	0.031	79.2	0.029
pN+	60	56.1		57.3	
GOLPH3					
Low	71	69.0	0.036	70.4	0.023
High	77	51.9		51.7	

Abbreviations: LARC, locally advanced rectal cancer; DFS, Disease free survival; OS, Overall survival; CEA, carcinoembryonic antigen; GOLPH3, Golgi phosphoprotein 3.

**Table 5:** Multivariate analysis of survival in LARC

Parameters	Hazard ratio	95% Confidence interval	*P* value
5-year DFS			
pT	2.311	1.230–5.124	0.023
pN	2.589	1.574–4.935	0.015
GOLPH3	2.624	1.235–6.541	0.009
5-year OS			
pT	2.435	0.967–5.952	0.066
pN	2.635	0.358–6.891	0.052
GOLPH3	2.354	1.237–6.152	0.039

Abbreviations: LARC, locally advanced rectal cancer; DFS, Disease free survival; OS, Overall survival; pT, pathological tumor stage; pN, pathological node stage; GOLPH3, Golgi phosphoprotein 3.

## DISCUSSION

This is the first study to assess the associations of the new oncogene-GOLPH3 and nCRT in rectal cancer. The study confirmed the high expression of GOLPH3 in LARC, and revealed that GOLPH3 is an independent predictive factor for efficacy of nCRT. The underlying mechanism related with mTOR was also demonstrated.

In the study, we demonstrated that 77 of the 148 cases (52.03%) had high expression, which was higher than the rate obtained for normal tissue specimens (11/87, 12.64%; *P* < 0.001). Scott et al. demonstrated that GOLPH3 was highly expressed in lung, ovarian, breast, and prostate cancers, as well as and melanoma, in 2009 [11]. Further studies indicated that GOLPH3 was highly expressed in malignant tumors, including breast cancer (expression rate, 51.6%), renal cancer (53.23%), pancreatic cancer (72.5%), and non-small cell lung cancer (71.7%), and closely related to clinical staging of tumor, also being a factor predicting poor prognosis [14–17]. Guo et al. [13] and Wang et al. [18] showed that GOLPH3 was highly expressed in colorectal cancer (53.2% and 43.1%, respectively), i.e. significantly higher than normal tissue rates (24.2% and 13.3%), in agreement with the current findings. Besides, Among the 77 patients with high GOLPH3 expression, 31 and 19 had local relapse and distant metastasis, respectively. The resulting rates were 2.58 and 2.71 fold those of the low expression group, with higher malignancy degree in the high expression group, corroborating findings by Guo et al. [13] and Wang et al. [18].

The reasons for therapy failure in rectal cancer include local relapse and distant metastasis. Interestingly, nCRT and TME surgery greatly decrease rectal cancer relapse [19, 20]. Total mesorectum excision is graded and qualified by the pathologist, and is a strong predictor of several outcomes. The results of Maslekar et al. confirmed that grade of mesorectun independently influenced both local and overall recurrences [21]. Meanwhile, Leite et al. revealed that the mesorectal score was an independent factor for local recurrence and disease-free survival [22]. Furthermore, Lino-Silva et al. used a two-grade system to determine the prognostic value of the mesorectum quality, and results suggested that inadequate mesorectum was correlated with R1/R2 resections, positive margins, and decrease survival, so the two-grade system (adequate, inadequate) was proposed [23]. Because of the adverse outcome of inadequate mesorectum patients, our study excluded this part.

However, sensitivity to nCRT of rectal cancer varies. Many factors affect the sensitivity of rectal cancer to nCRT, including tumor invasion depth, lymph node metastasis status and serum CEA level [24], in line with the current findings. However, identifying predictive gene markers of nCRT sensitivity not only predicts nCRT effects conveniently and efficiently, but also provides possible target genes for increasing nCRT sensitivity. Therefore, we assessed the associations of the new oncogene GOLPH3 and nCRT in LARC for the first time. In this study, single-factor analysis demonstrated that 44 of the 71 cases with low expression had tumor down-staging (*P* = 0.020), and 41 patients showed TRG3–4 after nCRT (*P* = 0.009). Multi-factor analysis showed that GOLPH3 was an independent predictive factor for TRG (OR = 3.952; CI 1.655–10.327, *P* = 0.026) and tumor down-staging (OR = 2.951; CI 1.523–11.324, *P* = 0.021) (Tables 2, 3), suggesting GOLPH3 could reliably and independently predict sensitivity to nCRT in rectal cancer.

As carcinogenic mechanism, Scott et al. reported that GOLPH3 could activate the mTOR pathway [11], and Buschamn et al. found it could activate DNA-dependent protein kinase (DNA-PK), leading to DNA damage [10]. The yeast mTOR isomer (TOR) activates the Vps35 subunit of the retromer transport complex and regulates endocytosis. It was found that GOLPH3 could also activate Vps35 to increase mTOR activity. Furthermore, it improves various exogenous signals, and regulates the growth, proliferation and survival of cells [11, 25, 26]. Phosphorylation activation of the downstream protein p70 ribosomal S6 kinase (S6K) in the mTOR signaling pathway is closely related with copy number in the 5p13 fragment [6, 11, 27], suggesting GOLPH3 involvement in the tumor is related to the mTOR activity. Because GOLPH3 activates mTORC1 and mTORC2 pathways simultaneously [6, 11, 28], it could be deduced that if GOLPH3 expression is inhibited, mTOR down-stream signal pathways would be effectively decreased. Therefore, GOLPH3 could be used as an efficient target for tumor therapy. Besides, Scott et al. demonstrated that GOLPH3 could increase sensitivity to rapamycin [11]. In this study, GOLPH3 expression was found closely related to mTOR levels (*r* = 0.745), as shown by Figure 2. Whether the mTOR pathway is targeted by GOLPH3, with its inhibition reversing resistance to radiotherapy in rectal cancer needs to be addressed in future studies.

Single and multiple-factor analyses indicated that GOLPH3 was an independent prognosis factor for rectal cancer as listed in Tables 4, 5. Indeed, the patients with high expression had poor prognosis, with 5 year-survival rates lower compared with the low group (51.7% vs. 70.4%) (Figure 3). Guo et al. found a 5 year-survival rate of 48.6% in patients with high GOLPH3 expression, for 69.4% in the low group [13], corroborating our findings. However, Wang et al. showed that DFS and OS of patients with colorectal cancer under 5-Fu adjuvant chemotherapy were improved in patients with high GOLPH3 expression [18]. The authors showed that GOLPH3 overexpression promoted the apoptotic effect of 5-Fu and increased sensitivity to 5-Fu, which resulted in increased survival. It is the mechanism underlying GOLPH3 effect on adjuvant/neoadjuvant chemotherapy that likely the primary cause for the difference between these results.

However, some inherent limitations of this study might lead to biased results. First, the current study was a retrospective study and the number of patients in our study was limited. Second, whether a biopsy is representative of the whole tumor need to be investigated because of tumor heterogeneity.

In conclusion, this study demonstrated that GOLPH3 was highly expressed in rectal cancer, and could predict sensitivity to nCRT in LARC; effect was better in patients with low expression. In addition, GOLPH3 expression was closely related to mTOR expression, indicating that it might act through the mTOR pathway. Finally, GOLPH3 was shown to be an independent prognosis factor for rectal cancer. However, due to the limitations of the current study, these findings should be confirmed by further well-designed experiments.

## MATERIALS AND METHODS

### Patients and clinical assessment

Clinical and pathological characteristics of 163 cases with rectal cancer between January 2008 and December 2010 were retrospectively analyzed. Biopsy tissues were obtained by proctoscopy from the patients before therapy to confirm the pathological diagnosis of adenocarcinoma within 15 cm from the anal verge. Physical examination, CEA, routine blood test, chest enhancement CT, as well as abdomen and pelvic cavity enhancement CT were performed before therapy. Then, the patients were staged according to the AJCC criteria (Edition 7) [29]. Seven patients with distant metastasis and 5 others declining surgery after nCRT were excluded, as well as 3 cases who had insufficient tumor tissue samples. A total of 148 patients with rectal cancer in T3-T4/N+ stage receiving nCRT were included. They all had consistent basic characteristics, and provided signed informed consent. The study was approved by the Ethics Committee of Shandong Cancer Hospital.

### Multimodal treatment

All the rectal cancer cases received nCRT, followed by surgical treatment (TME). Specifically, whole pelvic radiotherapy was performed with DT = 50.4Gy/28 times (four fields of irradiation or 3D conformal radiotherapy). The concurrent chemotherapy included 5-Fu (5-fluorouracil) continuously pumped or capecitabine in combination with oxaliplatin. TME was performed at 4∼6 weeks after nCRT.

### Pathological assessment

Two pathologists blinded to clinical information analyzed the postoperative pathological results, respectively. Preoperative biopsy tissues and postoperative pathological tissues were staged according to the AJCC staging criteria to assess the tumor down-staging effect of nCRT. Furthermore, sensitivity to nCRT of the tumor was assessed according to Dworak et al. [30]. The standards were as follows: TRG0, no regression; TRG1, tumor-based with significant fibrosis and/or vasculopathy; TRG2, fibrosis change-based with very few tumor cells or tissues (easily observed); TRG3, very few tumor cells in fibrotic tissues (easily observed by microscopy); TRG4, no tumor cells in fibrotic tissues (complete regression). TRG3 and 4 were defined as a good response, while TRG 0, 1 and 2 reflected a poor response. TRG 4 represented pCR.

### Immunohistochemical analysis of GOLPH3 and mTOR

A total of 148 biopsy tumor tissue samples before nCRT therapy were collected. The paraffin embedded specimens were sliced in 5 μm thickness, and GOLPH3 and mTOR expression levels were assessed by immunohistochemistry. Tissue sections were dewaxed by xylene, and hydrated by a graded ethanol series and distilled water. After antigen retrieval, normal rabbit serum was added (ZSGB-Bio, China) for blocking at room temperature for 20 min. Then, rabbit anti GOLPH3 and mTOR antibodies (Abcam, Cambridge, UK) at 1:200 were used to stain the samples for 1 h at room temperature. Goat anti-rabbit secondary antibodies (ZSGB-Bio) were added for 15 min at 37°C. Signals were revealed by 3,3-DAB, and hematoxylin was used for counterstaining. Finally, the slices were dehydrated by a graded ethanol series and xylene, mounted and examined by microscopy.

Two pathologists assessed the results according to the GOLPH3 scoring standard, to determine the rate of GOLPH3 positive cells and staining intensity as follows: 0, no positive cells; 1, 1∼10% positive tumor cells; 2, 11∼35% positive tumor cells; 3, 36∼70% positive tumor cells; 4, > 70% positive tumor cells. Staining intensity was scored as follows: 0, no staining; 1, weak positive (slight yellow); 2, medium staining (claybank); 3, strong positive (sepia) [31].

Staining index for GOLPH3 expression was calculated by the two scoring systems, yielding possible scores of 0, 1, 2, 3, 4, 6, 9, and 12. Scores higher than 6 were considered high expression; those lower than 4 represented low expression [31]. To assess the relationship between mTOR and GOLPH3 levels, mTOR expression was assessed in a similar fashion.

### Follow-up

The enrolled patients were followed-up strictly, once every 3 months in the first 2 years, and every 6 months in the following years. Median follow-up time was 58 months. Follow-up parameters included physical examination, serum CEA level, peripheral blood count, chest X-ray, as well as abdomen and pelvic cavity enhancement CT or MRI. Colonoscopy was performed once yearly.

### Statistical analyses

For all the statistical analyses, spss 17.0 (SPSS Inc., Chicago, IL, USA) was used. The association of GOLPH3 status with clinicopathologic parameters (gender, age, differentiation, distance from anal verge, clinical tumor/node status, recurrence and distant metastasis) was assessed using the χ^2^-test. The Chi-squared test was also performed to assess the association between pathologic factors including GOLPH3 expression and tumor response. A multivariate stepwise logistic regression analysis was performed in order to determine the independent prediction of all variables that were significant in the univariate analysis. The associations between GOLPH3 and mTOR protein expression was analyzed by Chi-square test and correlation analysis. Overall survival (OS) was calculated from the initiation date of treatment to death from any causes or censored date of last contact for surviving patients. Disease free survival (DFS) was calculated from the initiation date of treatment to the date of any evidence of local or systemic cancer recurrence. The impact of the GOLPH3 status on OS and DFS was assessed by Kaplan-Meier method for the univariate survival analysis and Cox proportional hazards model for multivariate survival analysis. *P*-values < 0.05 or 95% were considered statistically significant differences.
